# Ultrasonic Vibration-Assisted Ball Burnishing Tool for a Lathe Characterized by Acoustic Emission and Vibratory Measurements

**DOI:** 10.3390/ma14195746

**Published:** 2021-10-01

**Authors:** Ismael Fernández-Osete, Aida Estevez-Urra, Eric Velázquez-Corral, David Valentin, Jordi Llumà, Ramón Jerez-Mesa, J. Antonio Travieso-Rodriguez

**Affiliations:** 1 Department of Mechanical Engineering, Universitat Politècnica de Catalunya, 08019 Barcelona, Spain; ismael.fernandez.osete@upc.edu (I.F.-O.); eric.velazquez.corral@upc.edu (E.V.-C.); antonio.travieso@upc.edu (J.A.T.-R.); 2 Department of Mechanical Engineering and Manufacturing, Universidad de Sevilla, 41092 Sevilla, Spain; aeurra@us.es; 3 Centre for Industrial Diagnostics and Fluid Dynamics, Universitat Politècnica de Catalunya, 08034 Barcelona, Spain; david.valentin@upc.edu; 4 Department of Science and Materials Engineering, Universitat Politècnica de Catalunya, 08019 Barcelona, Spain; jordi.lluma@upc.edu

**Keywords:** accelerometer, acoustic emission, ball burnishing, natural frequencies, operational deflection shape, piezoelectric, process monitoring, ultrasonic

## Abstract

This paper focuses on a resonant system used to induce a low-amplitude movement and ultrasonic frequency to complement a ball burnishing process on a lathe. The system was characterized through the combination of different techniques. A full vibratory characterization of this process was undertaken with the purpose of demonstrating that the mechanical system—composed of the tool and the machine—does not present resonance phenomena during the execution of the operation that could lead to eventual failure. This dynamic analysis validates the adequateness of the tool when attached to an NC lathe, which is important to guarantee its future implementation in actual manufacturing contexts. A further aim was to confirm that the system succeeds in transmitting an oscillating signal throughout the material lattice. To this end, different static and dynamic techniques that measure different vibration ranges—including impact tests, acoustic emission measurement, and vibration measurement—were combined. An operational deflection shape model was also constructed. Results demonstrate that the only high frequency appearing in the process originated in the tool. The process was not affected by the presence of vibration assistance, nor by the burnishing preload or feed levels. Furthermore, the frequency of the assisting ultrasonic vibration was characterized and no signal due to possible damage in the material of the specimens was detected. These results demonstrate the suitability of the new tool in the vibration-assisted ball burnishing process.

## 1. Introduction

In order to control several parameters of machining processes, different measurements (vibrations, energy consumption, airborne noise, acoustic emission, etc.) can be determined and processed using various signal-processing techniques.

Acoustic emission (AE) is one of the most frequently used measurements for this purpose. AE can be described as a set of elastic pressure waves generated by the rapid release of energy stored within a material. This energy dissipation is basically due to dislocation motion, phase transformations, friction, and crack formation or growth [1].

Different vibration and signal measurement techniques have been used in the past for the detection of failures in manufacturing processes [2]. Several authors [3,4,5,6,7] discuss how AE is related to the wear mechanisms of cutting tools. Pandiyan and Tjahjowidodo [8] applied dynamic measurements to establish the fault thresholds in grinding wheels under different conditions, while Lopes et al. [9] monitored the condition of a grinding wheel. Wang et al. [10] and Zanger et al. [11] studied the relationship between the AE and chip size.

The quality requirements of industrial products are constantly increasing and machined components are no exception. For this reason, ultrasonic-assisted tools are now used to improve surface quality. Hence, new methodologies are required to study the vibratory behavior of these kinds of tools, as described in the previous paragraph.

Referring to finishing operations, the British scientist Griffith concluded in 1921 that the strength of materials with isotropic properties was much lower (between 10 and 20 times) than could be predicted theoretically and that this is due to the lack of continuity of the material; that is, the existence of defects [12]. These defects occur in the process of obtaining the components (metallurgical defects) or in the production process due to geometric details. Defects of the surface layers of machined components are especially dangerous. In these surface layers, three properties are especially important: surface hardness, roughness, and compressive residual stresses.

Ball burnishing is one the most suitable processes with which to improve these properties [13,14]. This process consists of the plastic deformation of irregularities in the target surface through the application of a controlled force by a sphere [15]. In recent years, the technical world has witnessed the birth of vibration-assisted ball burnishing (VABB). In this technique, the ball that compresses the target surface is subjected to a high-frequency vibration (between 20 and 40 kHz) which, in turn, is transmitted to the target surface [16]. This vibration of the surface material produces a lowering of its yield limit—a phenomenon called acoustoplasticity [17]. As a result, plastic deformation of the material is achieved with forces lower than those that would be necessary without vibration assistance. Consequently, VABB provides better results than conventional or non-vibration-assisted ball burnishing (NVABB) [18].

Different systems have been used to enhance ball burnishing with vibrations in a variety of different machining processes [16,17,18]. Most of these systems, including the one studied in this paper, use a resonant system characterized by a low amplitude movement (between 3 and 30 µm) [19]. This system, which is described by Jerez-Mesa [20], applies a high-frequency electrical charge to a piezoelectric stack, causing it to undergo a deformation which is then transmitted to the ball. This device is called a sonotrode [21].

With regard to burnishing, there are few studies where the application of AE is so direct. Dornfeld and Liu [22] concluded that AE helps to reveal information about the frictional behavior of the ball burnishing process, as it has a strong correlation with the kinetic friction coefficient and the texture surface profile. Their work also concluded that AE demonstrates how the burnishing process can be divided into four stages from a dynamics perspective. Only in the first two can positive results during burnishing be obtained. Strömbergsson et al. [23] observed that monitoring AE parameters during the burnishing process to confirm that the operation has performed its intended function is highly beneficial. For example, inspection of an AE signal in root mean square (RMS) representation for 5 min demonstrated that the decrease in the coefficient of friction (COF) stagnated after a time, and that the tribological behavior did not remain stable. Therefore, investigators should be made aware of the effects of excessive wear of the burnishing ball and how these can affect the finishing results. Salahshoor and Guo [24] used AE to monitor the burnishing process on a magnesium-calcium alloy.

In addition to the limited studies regarding the application of AE to burnishing, some studies apply this technique to the diagnosis of possible faults in contacts between solid bodies in relative motion—a case to which burnishing is easily applicable due to the way it takes place, and which is therefore considered relevant as an antecedent to this paper [25]. Tandon et al. [26] highlighted the effectiveness of AE for detecting failures in contacts between ball bearings, and concluded that it can detect the transfer of particles from the wear of the two surfaces in contact. They also concluded that AE is more effective than vibration analysis as it can detect errors before they occur. Hase et al. [27] concluded that the frequency spectra of the AE signals measured during the tribological tests allowed them to determine the wear mechanism between the contact surfaces. Geng et al. [28] concluded that AE signals acquired at the highest sampling frequencies were more sensitive for the detection of the friction mechanisms between two contact surfaces than the evolution of the friction coefficient that characterizes this contact.

In this paper, a full vibration characterization of a ball burnishing process performed in a lathe is presented. This ball burnishing process was performed using a tool designed by the authors [29]. The main objective was to demonstrate that the machine and the tool do not present any resonance issues during their service that could result in possible hardware malfunctions. This dynamic analysis validates the suitability of the tool when it is attached to an NC lathe and is relevant to the eventual industrial users of the system. The designed tool is particularly intended for application in industries that manufacture elements with revolution symmetry that are subjected to high-cycle fatigue or in which a particular type of wear must be prevented. The adequateness of the system to transmit vibrations through the material is assessed.

A specific methodology was applied to validate the dynamic behavior of the tool by combining several techniques based on quantification of the normal and ultrasonic vibration ranges through static and dynamic measurements. In the static measurements, the frequency response functions of the tool were measured and, consequently, the natural frequencies were determined [30]. The dynamic measurements were used to characterize the burnishing process (vibration assisted or not) under operating conditions. In this case, acoustic emission was used to detect possible damage in the material during the VABB process.

The analyses included in the previous paragraph demonstrate the fundamental importance of the traditional techniques of static and dynamic vibration analysis as applied to the VABB process discussed here. The dynamic results derived from VABB applied to two different ferric alloys are described in order to evaluate different magnitudes under different burnishing conditions: two burnishing forces (90 N and 270 N) and the existence of vibration assistance (yes or no). The measured magnitudes were burnishing force, vibrations, and acoustic emission. This allowed us to characterize the process itself and the tool’s ability to transmit vibratory assistance, as well as to detect possible damage in the specimens produced by this process. From the vibration measurements, an operational deflection shape (ODS) exercise was also performed.

This research is novel as, despite the fact that the vibration-assisted ball burnishing process is not new, a new tool that is capable of carrying it out was analyzed. This tool has a series of characteristics that make it unique from those on the market. Additionally, the AE technique applied for characterization and verification of the influence of the vibration assistance did not produce any negative effects on the process results. No reference to the use of AE for this purpose was found in the reviewed literature.

## 2. Materials and Methods

### 2.1. Experimental Setup

In order to characterize the dynamic behavior of the machine-tool-part setup, two types of tests were performed: impact tests under static condition; and vibrations, forces, and acoustic emission monitoring under running (dynamic) conditions of the burnishing system. The ball burnishing tool used was a prototype designed and patented by the authors [29]. It can be used in both NVABB and VABB processes. The frequency of its ultrasonic vibration is 40 kHz. All tests were performed in a PINACHO SE 200 × 1000 mm CNC lathe (Pinacho CNC, Guipuzcoa, Spain). The specimens were fixed between a self-centering three-jaw chuck plate and the point.

Different burnishing parameters were taken into account in this study: specimen material (C45 steel, EN 10020:2000, and GJL250 grey cast iron, EN 1560:2011), burnishing force (90 N and 270 N) and ultrasonic vibration assistance (yes or no). The specimens were previously machined. Table 1 presents their initial and final dimensions, the cutting parameters, and the measured roughness. No cutting fluids were used in the machining.

**Figure 1:**
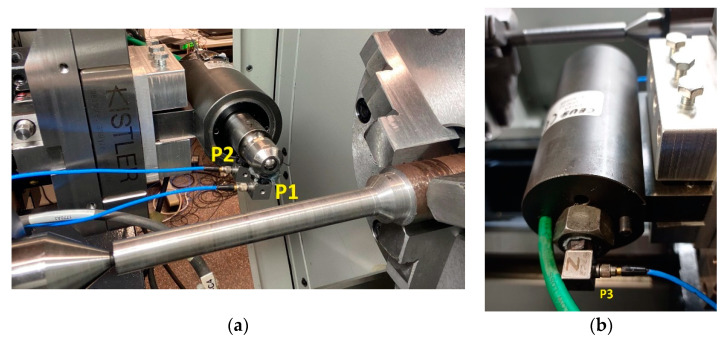
Triaxial accelerometers installed in the tool: (**a**) frontal view; (**b**) rear view.

Different accelerometers were used. Three triaxial accelerometers were installed in the burnishing tool in order to study its vibrating behavior. Two were mounted in the frontal part near the burnishing ball (positions P1 and P2 of Figure 1a), and the third was mounted in the opposite part (position P3 of Figure 1b). The characteristics of these accelerometers are presented in Table 2. The measurement directions of the accelerometers correspond to the burnishing feed (X), vertical direction (Y), and direction of the burnishing force (Z).

**Figure 2:**
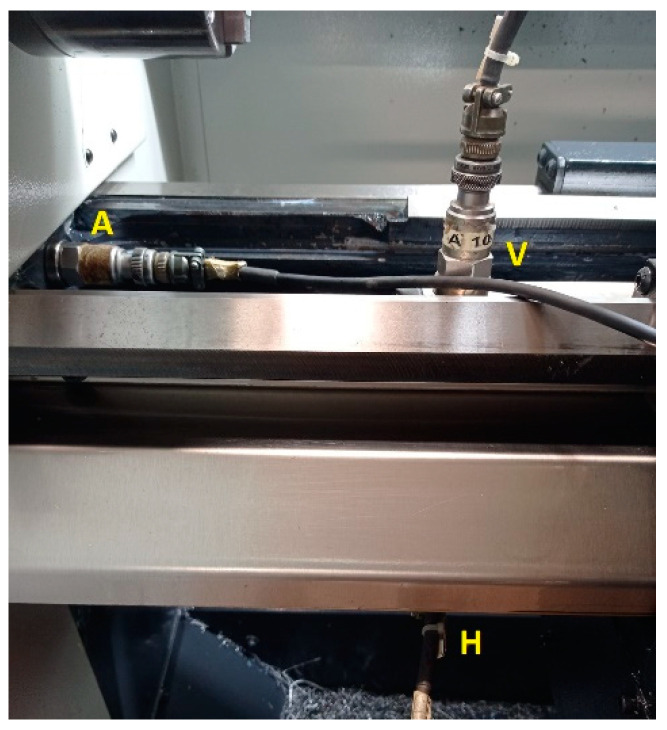
Accelerometers mounted on the lathe bed.

Three monoaxial accelerometers were installed in the lathe bed in order to determine the vibrational transmissibility from the machine during the burnishing process. Figure 2 depicts these accelerometers and their measurement directions: A (axial according to the specimen rotation), V (vertical), and H (horizontal). The characteristics of these accelerometers are presented in Table 2.

The monitoring criteria were selected taking the following into account:

- The maximum monitoring frequency allowed by the instrumentation should be applied.
- All mechanical and electrical phenomena that occur during the process should be monitored and recorded.

### 2.2. Burnishing Force Monitoring

The compressive deformation of the spring installed inside the tool-holder is linearly related to the force transmitted by the burnishing tool to the target surface [20]. In turning processes, it is linearly related to the penetration of the tool in the direction of the depth of the pass. The nominal burnishing forces of the tests were 90 N and 270 N for the steel specimen and only 90 N for the grey cast iron, as 270 N is an excessive load for this material.

**Figure 3:**
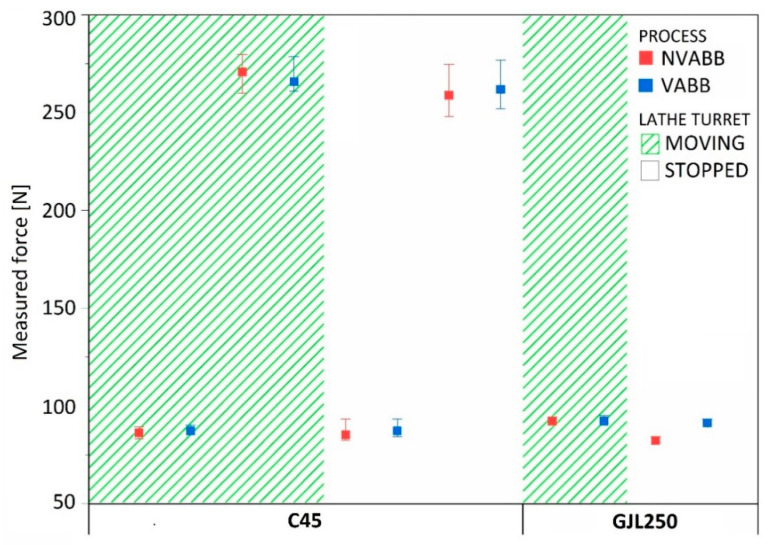
Average, maximum, and minimum forces recorded during the tests.

The force was monitored by a KITSLER 9129AA dynamometer (Kistler, Winterthur, Switzerland) adapted to the lathe holder where the tool was mounted, as seen in Figure 1a. The force signal was conditioned by a KITSLER 5070A12100 amplifier (Kistler, Winterthur, Switzerland) Burnishing forces were acquired in impact tests under static conditions (lathe turret stopped) and in measurements under operating conditions (lathe turret moving) of the burnishing system (Figure 3).

### 2.3. Impact Tests

Impulse excitation was used to determine the natural frequencies of the tool under different conditions. Impacts were carried out with an impact hammer (KISTLER 9722A2000) (Kistler, Winterthur, Switzerland) with steel tip (9902A) (Kistler, Winterthur, Switzerland) Its maximum frequency was 9.3 kHz and its maximum force was 11 kN. These characteristics were deemed suitable as, according to a previous study [30], the natural frequencies of a similar tool were lower than 5 kHz.

**Figure 4:**
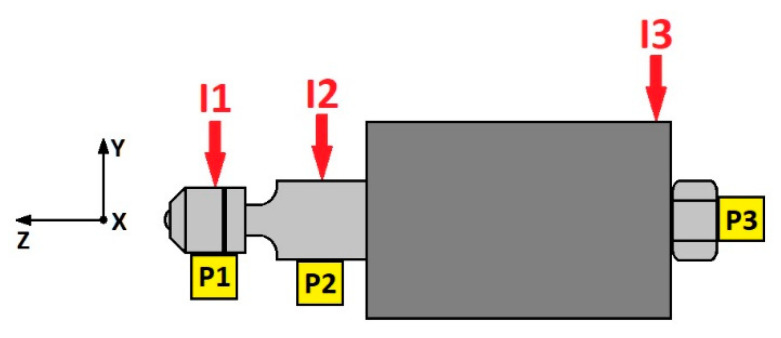
Lateral view of the impact points on the tool.

Impacts were performed at three tool points in the vertical direction (points I1, I2, and I3) and vibrations were measured at three points (P1, P2, and P3) in three directions, as seen in Figure 4. The conditions under which the impacts were carried out were as follows:

- Tool installed it its holder without any contact with the specimen (free tool).
- Tool in contact with the specimen and forces between them of 90 N or 270 N, for the steel specimen, and a force of 90 N for the grey cast iron. These conditions were repeated with the tool in two positions: one near the plate and the other near the point.

In total, 15 impact tests were performed. The vibration assistance was not activated during the impact tests as a previous work [31] demonstrated that the ultrasonic vibration of the assistance does not affect a tool’s natural frequency.

The acquisition and further analysis of the vibration signals were performed with a 3053-B-120 analyzer (Brüel & Kjær, Nærum, Denmark) and the PULSE Reflex software (version 2.3), respectively. Ten channels were defined: three for each triaxial accelerometer and one for the impact hammer.

### 2.4. Vibration Monitoring during the Burnishing Process

Different burnishing tests with different forces were performed in order to characterize the machine-tool-specimen setup during the burnishing process. The burnishing forces used were 90 N and 270 N for the C45 steel and 90 N for the GLJ250 grey cast iron. The second variable was the presence (VABB) or absence (NVABB) of ultrasonic vibration in the tool. The burnishing speed was 2.33 m/min and the feed was 0.15 mm/revolution. The burnished length of each test was 10 mm, except for some tests that were carried out without feed for 3 min.

**Figure 5:**
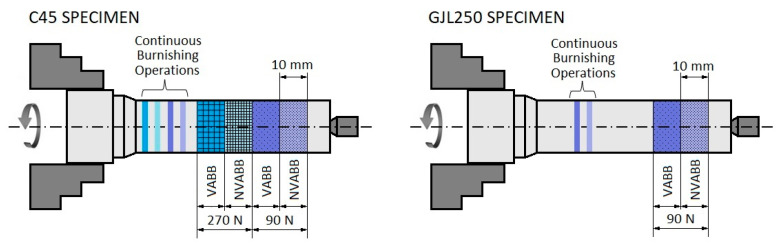
Burnishing test areas.

The layout of these tests are presented in Figure 5 for both the C45 and GJL250 specimens.

From these measurements, an ODS of the tool was obtained. ODS is a vibration analysis tool that allows for the determination of the deflection of a component or structure under real operating conditions. Vibration time histories are recorded under operating conditions and, by applying the Fourier transform to these recordings, vibration level versus frequency is determined at different points. A system’s wire frame model can then be animated in order to demonstrate the movement at each measured point and at each frequency [32].

### 2.5. Acoustic Emission Measurements during the Burnishing Process

As previously explained, acoustic emission waves are high-frequency waves (in the ultrasonic frequency band) generated when any kind of damage is produced in a material. During manufacturing processes, different acoustic emission signals are usually emitted by machined parts as a consequence of the damage produced in them. For the burnishing process presented in this paper, the eventual presence of acoustic emission events was explored as previous studies have validated the application of this this technique to the characterization of the process itself.

To this end, a Vallen acoustic emission sensor, model VS700-D (Vallen Systeme, Wolfratshausen, Germany), was installed in the tool holder. A Vallen preamplifier, model AEP4, a Vallen AMSY5 acoustic emission system (Vallen Systeme, Wolfratshausen, Germany), and Vallen acquisition software (Vallen Systeme, Wolfratshausen, Germany) were used for conditioning and recording of the acoustic emission signals. The sampling frequency of the acquisition was 625,000 samples/s.

## 3. Results and Discussion

### 3.1. Impact Tests

Impacts performed at point I3 (Figure 4) did not provide any valid information because the base housing was disconnected from the tool in order to ensure that the ultrasonic vibrations were transmitted to the lathe [29].

According to the impact position, the best responses always corresponded to the vertical direction (Y), that is, the impact direction. Frequency response functions (FRF) of point P3 revealed poor coherence between excitation and response.

**Figure 6:**
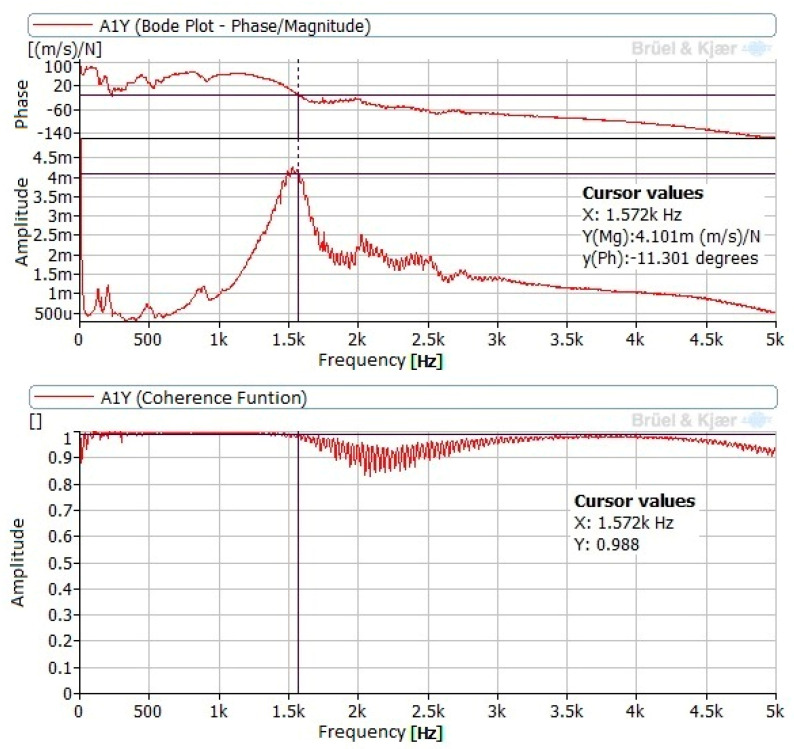
FRF obtained at P1 with impact at I2.

Signals measured at points P1 and P2, corresponding to the tests performed without contact between tool and specimen, revealed a natural frequency of around 1.5 kHz. Figure 6 presents the FRF and the coherence function corresponding to the response at point P1 with excitation at I2. Some low peaks also appear around 500 Hz and 900 Hz. The band between 2 and 3 kHz is also noticeable but its coherence is poor.

**Figure 7:**
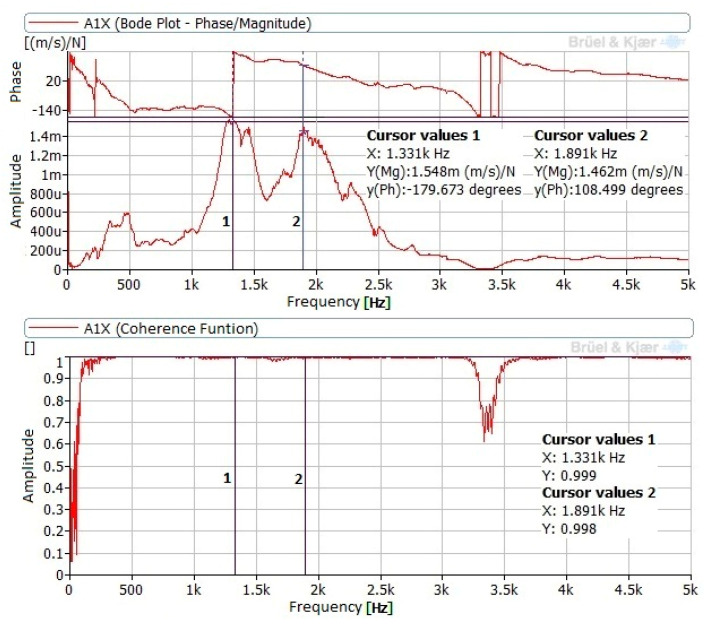
FRF obtained at P1 with impact at I1, with a load of 270 N.

In the tests performed with contact between the tool and specimen, no differences were noted between the different test conditions (force and tool position). Consequently, the load and tool position did not affect results. In these cases, natural frequencies in the signals measured at point P3 are noticeable. New components in the band between 1.2 and 2.8 kHz appeared. The amplitudes were lower than those obtained in measurements without contact between the tool and specimen. Figure 7 presents the FRF and coherence corresponding to response at P1 and impact at I1, in the position near to the lathe point and with a load of 90 N.

Natural frequencies were always much lower than the assisting frequency, that is, 40 kHz constituted just 5% of this magnitude.

### 3.2. Vibration Monitoring during the Burnishing Process

Measurement signals acquired by tool accelerometers (Figure 1) and test bed accelerometers (Figure 2) during burnishing processes were processed and analyzed in time and frequency domains. The maximum analysis frequency was 4 kHz. Spectra are presented, in some cases, up to 2 kHz as no components appeared at higher frequencies.

**Figure 8:**
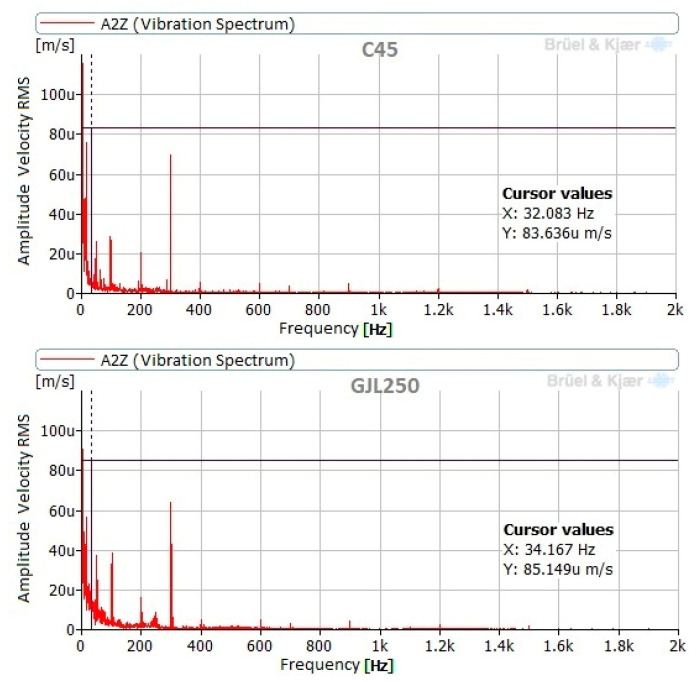
Vibration spectra measured at point 2, in Z direction (direction of the burnishing force), with a NVABB force of 90 N.

The vibration behavior of the tool during the burnishing process was similar for both materials under all burnishing conditions. Components at 15.8 Hz and 32.1 Hz were noticeable when burnishing C45, while burnishing GJL250 resulted in noticeable components at 17 Hz and 34.2 Hz. This difference is due to the slightly different burnishing speeds and the relation between these components and the mechanical and electrical operation of the lathe. In all cases, these components are very small (µm/s RMS). According to the ISO 20816 [33] for vibrations in machines, the highest allowable vibration level is 0.28 mm/s RMS: all of the measured levels were considerably lower. Figure 8 presents the spectra in the Z direction (direction of burnishing force) for both materials with a burnishing force of 90 N and without vibration assistance.

In tests without vibration assistance, the amplitudes corresponding to points P1 and P2 in burnishing force direction (Z) were higher than amplitudes corresponding to the other two directions: burnishing feed direction (X) and vertical direction (Y). This result can be justified as these points are at the end of the tool and receive all the effort of the burnishing operation. In the case of C45, the amplitudes at point P3 were greater than at points P1 and P2, while in GJL250 the amplitudes at the three points were similar. At point P3, the highest amplitude was in the Y (vertical) direction, which may be due to point P3 being in the distal part of the tool at the time of burnishing, as shown in Figure 1b.

**Figure 9:**
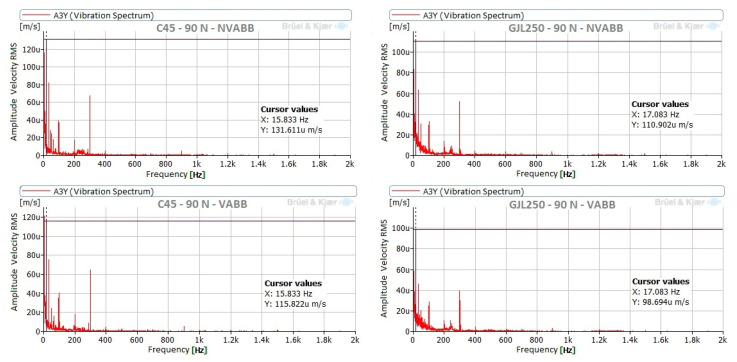
Comparison of spectra of both materials, using VABB and NVABB, measured at point P3, in Y (vertical) direction, and with a burnishing force of 90 N.

Figure 9 compares the spectra, measured with a burnishing force of 90 N for both materials, with and without vibration assistance, at point P3. This figure shows the Y-axis only because all directions demonstrated the same frequency peaks with very similar amplitudes. Additionally, signals measured without vibration assistance had similar behavior to those with vibration assistance.

**Figure 10:**
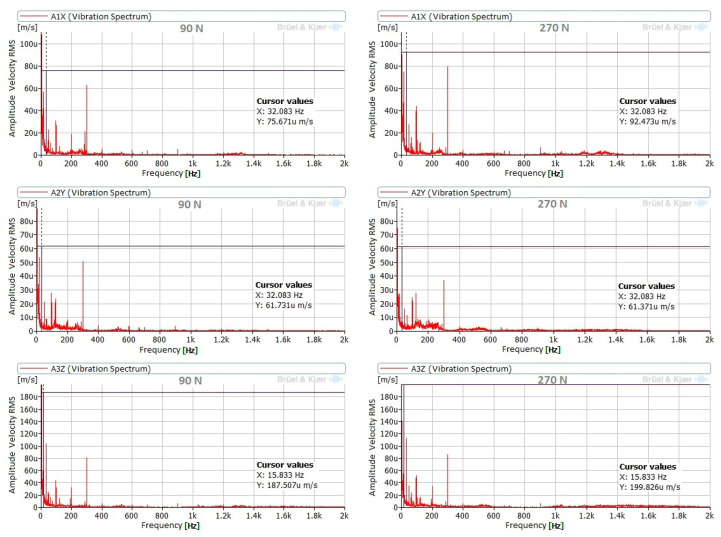
Comparison of spectra of C45 with burnishing forces of 90 N and 270 N, without vibration assistance.

Two burnishing forces were used in tests with C45 steel. No differences between the signals monitored under these conditions were noted. To demonstrate this, Figure 10 presents the spectra corresponding to different points and measurement directions. All of these spectra correspond to measurements without vibration assistance.

**Figure 11:**
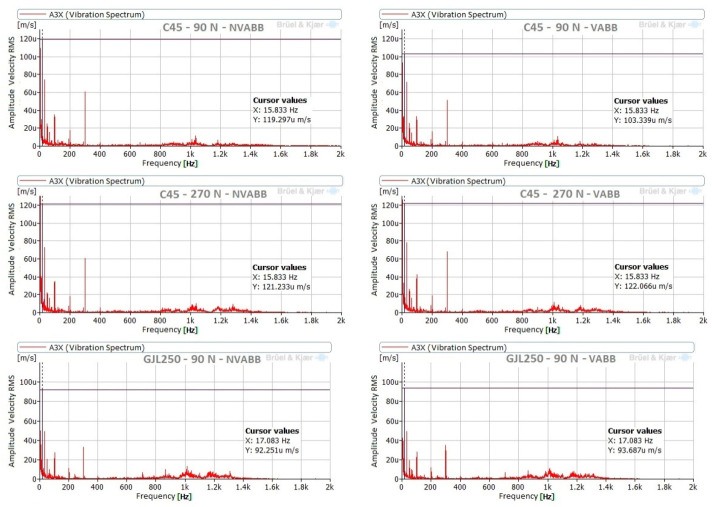
Measurements in burnishing without feed.

Additionally, measurements without burnishing feed were carried out for both materials, under the same conditions previously specified. The duration of each of these measurements was 3 min. Analysis of these tests reveals similar results to those obtained with burnishing feed. The same frequency peaks appeared, with very small amplitudes. The only difference was the possible excitation of natural frequencies in the range between 800 Hz and 1400 Hz, but with very low amplitudes. Figure 11 presents an example of these signals.

The vibration measurements in the lathe test bed show normal behavior of the machine. Components that interfere with those obtained in the tool were not noted, but a possible excitation of the natural frequencies between 800 Hz and 1400 Hz was noticeable, but with very low amplitudes.

**Figure 12:**
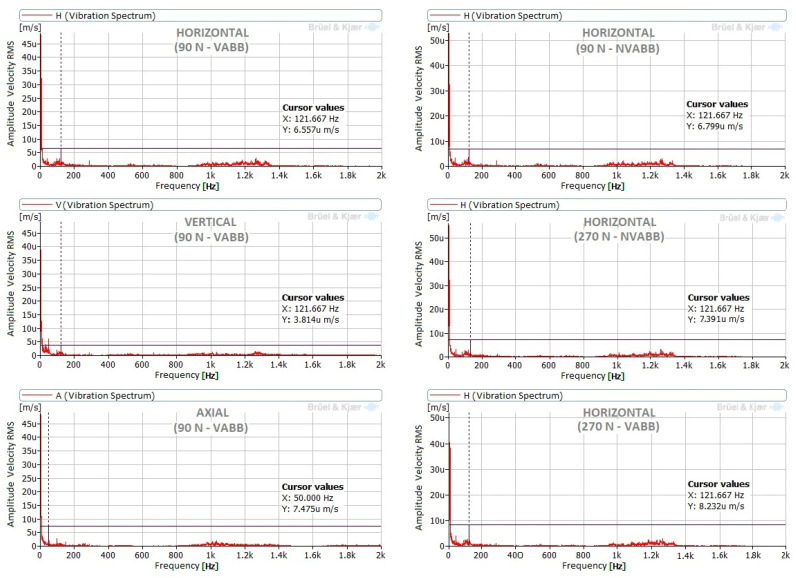
Spectra measured in lathe test bed at different loads and different measurement positions.

Figure 12 presents signals measured in the lathe test bed during the burnishing of the steel specimen. Comparing the signals measured with vibration assistance in the horizontal (H), vertical (V), and axial (A) measurement positions, a very similar behavior was noticeable between them, and the amplitudes were again very low. Via inspection of the non-vibration-assisted spectra measured in the horizontal direction at burnishing forces of 90 N and 270 N (Figure 12), no differences appeared between them; consequently, the burnishing force did not affect the horizontal vibrations of the lathe test bed. Additionally, no differences appeared between horizontal spectra with or without vibration assistance at both burnishing forces in Figure 12; therefore, there was no influence of the vibrating assistance in the lathe test bed. This is expected according to the tool design, lathe rigidity, and the test bed points where the measurements were performed.

### 3.3. Acoustic Emission Monitoring during the Burnishing Process

**Figure 13:**
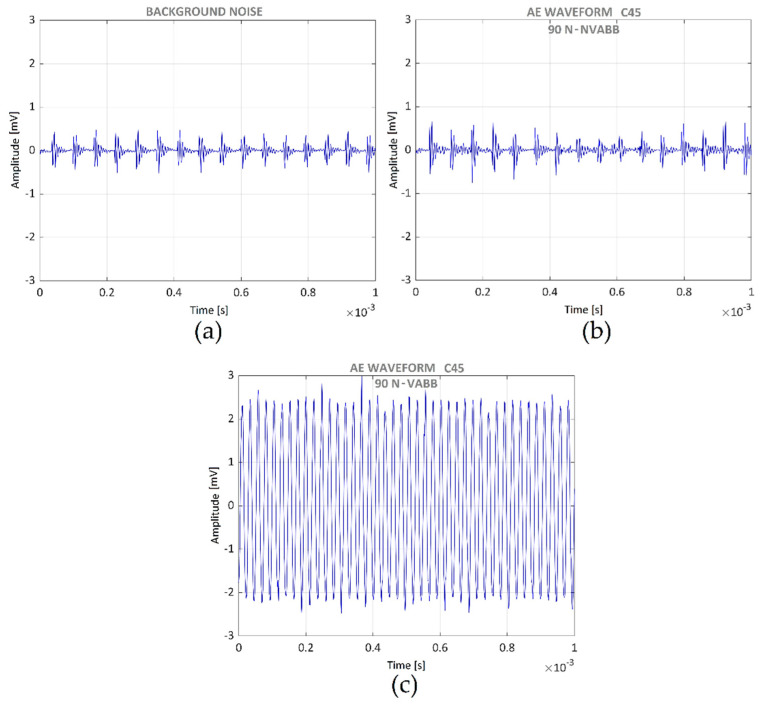
AE time histories of C45 under different conditions: (**a**) background noise; (**b**) NVABB; (**c**) VABB.

Figure 13 presents acoustic emission time histories for different processes. Figure 13a presents the background noise. Figure 13b presents the acoustic emission signal during the burnishing process of the C45 steel specimen with a burnishing force of 90 N, without vibration assistance. Finally, Figure 13c presents the C45 burnishing with a burnishing force of 90 N, with vibration assistance. Figure 13a and Figure 13b are very similar; therefore, the burnishing process without vibration assistance did not produce any acoustic emission. However, a clearly noticeable signal appears in Figure 13c, corresponding to the vibration-assisted burnishing process. This is due to the acoustic emission sensor detecting the assisting vibration signal. No acoustic emission apart from that produced by the vibration assistance appeared; consequently, the ball burnishing process did not produce any damage in the material.

**Figure 14:**
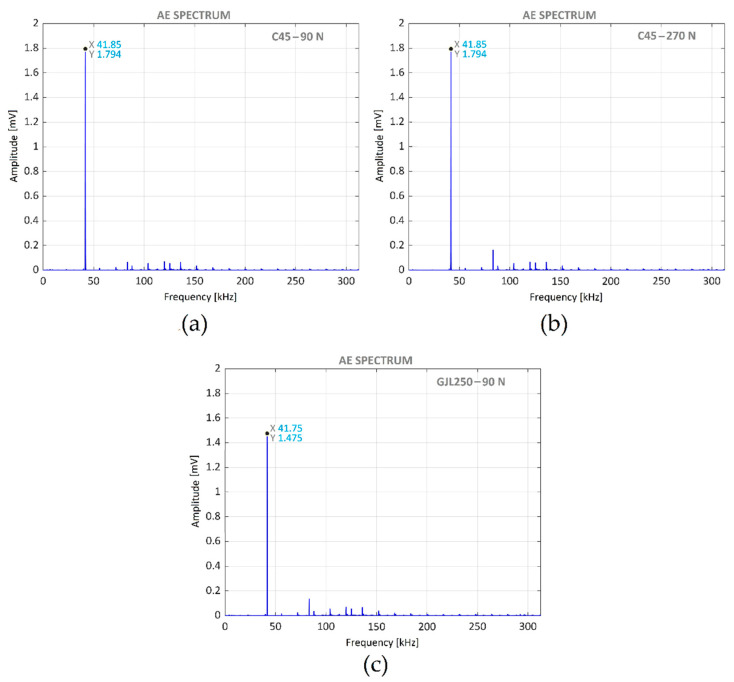
AE spectra corresponding to different VABB: (**a**) C45 steel with 90 N; (**b**) C45 steel with 270 N; (**c**) GJL250 cast iron with 90 N.

Figure 14 presents the AE spectra corresponding to the VABB of both materials, burnished with different forces: (a) C45 steel specimen with 90 N, (b) C45 steel specimen with 270 N, and (c) GJL250 grey cast iron specimen with 90 N. In all figures, the frequency corresponding to the vibration assistance is the only noticeable peak. This is consistent with previous findings [20].

The vibration assisting frequency was very stable. Its variation of 100 Hz corresponds to 0.25% of the frequency. This level remained the same for both VABB of C45 steel; thus, the burnishing force did not influence it. The vibration level, in the case of GJL250 VABB, was lower those of C45 due to the high GJL250 internal damping [34].

### 3.4. Operating Deflection Shape

**Figure 15:**
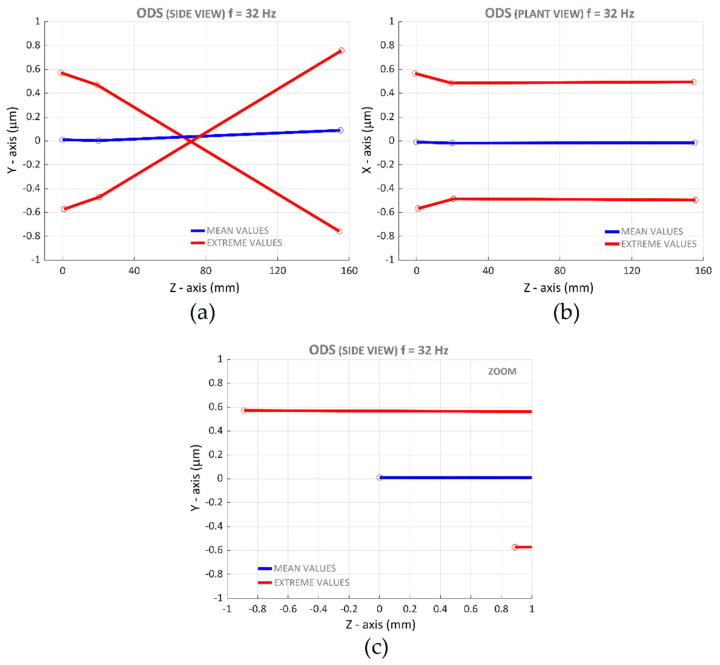
ODS of the tool at 32 Hz: (**a**) lateral view; (**b**) plant view; (**c**) zoom of lateral view.

According to the method presented in [35], an ODS of the tool in the burnishing process of C45 with a force of 90 N and without vibration assistance was performed to analyze the tool rigidity. A frequency of 32 Hz was selected as it had a noticeable displacement and an acceptable background noise. Figure 15 presents the ODS. Figure 15a is a lateral view and depicts the movement in the vertical direction. Red lines correspond to extreme positions and blue lines correspond to the mean position. A rotatory movement around the center of the tool is clearly noticeable, with an amplitude at the extremes of around 0.6 µm. Figure 15b presents the plant view. A translation movement in the burnishing feed direction of about 0.5 µm amplitude is also noticeable. In order to determine the movement in the axis of the tool direction, a zoom around the zero-point was performed (Figure 15c) and a displacement of about 0.9 µm was detected.

The methodology proposed in this paper (joining vibratory analysis with AE) allows one to extend the range of frequencies up to several hundred kHz. This approach is suitable for any machine-tool-part setup with different rigidities, configurations, and designs. Therefore, this may be used in the near future to characterize tools used in ultrasonic vibration-assisted machining operations such as those presented in [36]. Unfortunately, this methodology does not allow one to quantify the amplitude of the vibration in the AE range.

## 4. Conclusions

The conclusions of this paper are clustered according to the test through which they were obtained.

**Impact tests**

No natural frequencies higher than 2 kHz were noted (about 5% of 40 kHz, the vibration assisting frequency); consequently, the high frequency that appeared in the process was only that of the tool.

The structural behavior of the tool was affected by the contact between tool and specimen but was not affected by their contact force value.

**Vibration measurements**

The material of the workpieces, the vibration assistance, the burnishing force, and the feed movement did not affect the frequency spectra measured.

Spectrum lines appeared in the frequency range 800–1400 Hz during the burnishing process at the rear point and in the lathe bed with much lower amplitudes than those considered to be standard for a new machine, in accordance with the operation deflection shape results.

The vibration levels measured in the tool axis had higher amplitudes than those corresponding to the other directions, due to the burnishing force.

The amplitudes of the signals measured in the rear of the tool were slightly higher than those measured at the frontal part.

**Acoustic emission measurements**

The only signal detected was the ultrasonic vibration assistance, which permits the frequency characterization of the assisting ultrasonic vibration.

Any signal due to possible damage in the material of the specimens was detected.

## Figures and Tables

**Table 1:** Cutting parameters and specimen dimensions (initial and final).

Material	Initial Dimensions		Cutting Parameters			Final Dimensions		
	D [mm]	L [mm]	Cutting Speed [m/min]	Feed [mm/rev]	Cutting Depth [mm]	D [mm]	L [mm]	Roughness Ra [μm]
C45	15	133	70.7	0.15	0.2	14.8	133	1.187
GJL250	15	185	29.4	0.15	0.4	14.0	185	2.310

**Table 2:** Accelerometers used for the measurements.

Measurement Position	Accelerometer	Frequency Range [Hz]
Tool (P1, P2 y P3)	PCB 356A32/NC	1 ÷ 4000 ± 5% 0.7 ÷ 5000 ± 10%
Lathe bed, directions A, V y H	KISTLER Type 8752A50	0.5 ÷ 5000 ± 5%

## Data Availability

Not applicable.
